# Positive youth development and observed athlete behavior in recreational sport

**DOI:** 10.1371/journal.pone.0191936

**Published:** 2018-01-30

**Authors:** Matthew Vierimaa, Mark W. Bruner, Jean Côté

**Affiliations:** 1 Department of Kinesiology and Health Science, Utah State University, Logan, Utah, United States of America; 2 Schulich School of Education, Nipissing University, North Bay, Ontario, Canada; 3 School of Kinesiology and Health Studies, Queen’s University, Kingston, Ontario, Canada; UNITED STATES

## Abstract

**Objectives:**

Competence, confidence, connection, and character are regarded as outcomes of positive youth development (PYD) in sport. However, the specific athlete behaviors associated with different PYD profiles are not well understood. Thus, the purpose of this study was to investigate the relationship between athletes’ observed behavior during sport competitions and their perceptions of PYD outcomes.

**Design:**

Cross-sectional study with systematic behavioral observation.

**Method:**

Sixty-seven youth athletes were observed during basketball games near the end of their season, and the content of their behavior was systematically coded. Athletes also completed measures of the 4 Cs (competence, confidence connection, and character). A person-centered analysis approach was used to examine the relationship between PYD profiles and observed behavior.

**Results:**

A cluster analysis identified two homogenous groups of athletes characterized by relatively high and low perceptions of confidence, connection, and character. A MANCOVA revealed that after controlling for gender and years of playing experience, the high Cs group engaged in more frequent sport communication with their coaches.

**Conclusions:**

Results re-affirm the critical role that coaches play in the developmental experiences of young athletes, and highlight the importance of contextual factors of the youth sport environment.

## Introduction

Positive youth development (PYD) is a strength-based perspective that views youth as resources to be developed, rather than problems to be solved [1]. Essentially, the PYD perspective contends that all youth have personal strengths that can flourish and be promoted (e.g., [2]). The PYD approach began in developmental psychology approximately 20 years ago, and the majority of studies have investigated how youth’s participation in various forms of extra-curricular activities can influence important developmental outcomes (e.g., [3]). Researchers have suggested that effective PYD programs tend to be characterized by the provision of leadership opportunities, emphasis on the development of personal and life skills, sustained and caring youth-adult relationships, and a supportive and empowering environment [4–5]. Some have argued that organized sport may be a particularly fruitful context for the development of PYD [6–7]. As a result, the PYD approach has gained considerable popularity among sport researchers over the past decade (see [8–9] for reviews), leading to a proliferation of research across sport contexts using different conceptual frameworks.

One of the most dominant PYD frameworks across both developmental and sport psychology is the 5 Cs, popularized by Lerner and colleagues [3]. Lerner et al. posit that PYD occurs when youth exhibit growth in five distinct areas: Competence, confidence, connection, character, and caring. As youth develop in these five key areas over time, this can ultimately lead to a sixth C—contribution, whereby youth become thriving members of society who contribute to themselves, their families, and communities [10]. Following a review of the sport literature, Côté and colleagues [11] advocated for combining character and caring when studying PYD in a sport context, due to a lack of differentiation among these constructs in the extant sport literature. The resultant 4 Cs mirror the framework’s original conceptualization [12], which was expanded by Lerner and colleagues [3] using a similar review process to that of Côté and colleagues [11]. Using this sport-specific 4 Cs framework, Vierimaa and colleagues [13] conducted a subsequent review of literature and proposed a measurement toolkit using existing instruments, which assess the manifestation of the 4 Cs within a sport context. Based upon this approach [11, 13], each of the 4 Cs can be defined as follows: Competence reflects athletes’ skill level or ability in a given sport; confidence refers to athletes’ belief in their abilities to be successful in a given sport; connection is an umbrella term which comprises quality relationships among the social actors in a sport environment (e.g., coaches, teammates, etc.); finally, character refers to respect, responsibility, and ultimately engaging in prosocial behaviors and avoiding antisocial behaviors.

Recent studies have begun to apply this 4 Cs toolkit in sport research, demonstrating its utility to measure changes in PYD outcomes over time. Specifically, these studies have investigated the link between these PYD outcomes and observed coach behavior using systematic observation. For example, Allan and Côté [14] studied the relationship between the emotional tone of coaches’ behavior and athletes’ perceptions of the 4 Cs. The results of their study found that athletes of coaches who were calm and inquisitive reported more frequent prosocial behavior and less antisocial behavior toward opponents than athletes of coaches who conveyed a more negative and intense emotional tone. Erickson and Côté [15] adopted a longitudinal approach in their investigation of the intervention tone of coaches’ behavior in relation to athletes’ developmental trajectories over the course of a season. Interestingly, Erickson and Côté found that coaches interacted most often with athletes who scored the lowest on the 4 Cs. These studies provide critical insight into the important role of coaches’ on PYD in sport. However, we also know that youth’s sport experiences and development are shaped by the differential effects of multiple social agents (e.g., [16]), rather than the coach alone. Thus, there is a need to better understand how the full spectrum of athletes’ social interactions are linked with PYD outcomes.

The research on observed athlete behavior, specifically, is scant in comparison to the sizeable number of studies on coach behavior. However, there exists great potential in applying systematic observation to the study of athlete behavior in youth sport [17]. Several studies have examined observed athlete behavior in relation to performance outcomes (e.g., [18–19]), while others have recently begun to explore how observed athlete behavior is associated with PYD outcomes in different sport contexts. In their study on social status (i.e., connection) and athlete behavior among competitive adolescent volleyball players, Vierimaa and Côté [20] found that lower status athletes less frequently engaged in interactions with their teammates and coaches than their higher status peers. Erickson and Côté [21] studied interpersonal interactions in an informal sport play setting (i.e., recreational drop-in basketball games) and found that athletes with greater perceptions of competence tended to take on a leadership role and spent more time engaged with their peers in organizational behaviors. Overall, these two studies highlight the utility of using systematic observation to uncover the behavioral manifestation of PYD in sport. However, one must also remember that social interactions are constrained by the nature of the sport activity and environment in which they take place. Erickson and Côté [21] focused on peer interactions exclusively due to the nature of informal sport play, while Vierimaa and Côté [20] examined athletes’ interactions with both coaches and peers during highly structured competitive volleyball training sessions. Organized competition represents an important middle ground between these two contexts, as it is organized and includes the presence of many key social agents (e.g., coaches, teammates, opponents), but it can be more unpredictable than training sessions, and thus researchers may be more likely to observe salient social interactions that unfold in the heat of the moment that may not otherwise occur during training.

Thus, the purpose of this exploratory cross-sectional study was to investigate the relationship between athletes’ observed behavior during sport competitions and perceptions of PYD outcomes (i.e., the 4 Cs). Specifically, we aimed to uncover differential PYD profiles based on athletes’ responses on measures of the 4 Cs, and subsequently examine observed behavioral differences across these groups, in essence investigating the behavioral manifestation of PYD during sport competitions. Given the exploratory nature of this study and limited existing empirical evidence, no specific hypotheses were put forth.

## Methods

### Participants

Participants for the present study were 67 athletes and 20 head coaches from 20 teams in a single recreational basketball league in Ontario, Canada. Athletes ranged in age from 11–15 (*Mean*, *M* = 12.42; *Standard Deviation*, *SD* = 1.29), were predominantly male (68.7%), and had between 1–9 years of previous basketball playing experience (*M* = 2.73; *SD* = 1.96). Athletes were spread across three divisions: girls aged 11–14 (*k* = 7; *n* = 21); boys aged 11–12 (*k* = 8; *n* = 25); and boys aged 13–15 (*k* = 5; *n* = 21). Nine of the head coaches were female and 11 were male. The head coaches were between 24–59 years of age (*M = 35*.*31; SD* = 13.70) and had between 1–35 years of coaching experience (*M* = 8.46; *SD* = 11.38). Apart from two female coaches in the boys’ 11–12 year old division, all other coaches coached same sex athletes. All study participants and their parents provided active written consent prior to data collection. The study procedures were approved by the general research ethics review board at Queen’s University.

The basketball league is recreational in nature, and aside from a single practice at the beginning of the season, participants’ involvement is entirely made up of weekly games. Despite the competitive nature (e.g., scores are kept) of these weekly competitions, no long-term competitive elements are emphasized (e.g., standings, playoffs). Rather, all players receive equal playing time and the league strives to ensure that all players have fun, regardless of ability level. Additionally, the league is entirely volunteer-run and attracts a diverse mix of local youth by virtue of its low cost ($10 registration fee).

### Procedure

All of the participants’ teams were observed at two time points during the last month of their season in February and March of 2015. At each time point, all of the participants’ teams were audio and video recorded using two high-definition video cameras and a parabolic microphone. One camera was set up on a tripod with a static wide-angle perspective to capture both team benches. The other camera was located on the sidelines at center court and actively tracked the on-court action during play. The parabolic microphone was used to supplement the cameras’ built-in microphones to aid in capturing athletes’ verbalizations. The first time point served as pilot data and to acclimate the participants and coaches to the presence of the research team and equipment, while audio and video recorded during the second time point was retained for analysis [14, 20]. Immediately following the second time point, all of the participating athletes completed a battery of questionnaires that measured the 4 Cs (i.e., competence, confidence, connection, and character).

### Measures

#### 4 Cs

Athlete outcomes were measured using the 4 Cs toolkit, which is comprised of instruments that assess each of the 4 Cs: competence, confidence, connection, and character [13]. This toolkit was developed through a review of the sport literature and represents a collection of previously validated instruments that measure youths’ perceptions of the 4 Cs within a sport context. Participants were instructed to base their responses on their present team environment. As a whole, the toolkit has been applied in past research with youth soccer [14] and volleyball participants [20]. For further discussion of the selection of these individual instruments and their psychometric properties, see [13, 15].

#### Competence

Athletes’ perceptions of their competence in sport was measured using the Sport Competence Inventory (SCI; [13]), which expanded upon a single-item measure originally developed by Causgrove Dunn, Dunn, and Bayduza [22]. The SCI measures athletes’ self-perceptions of their competence in sport using three items that assess technical, tactical, and physical skills. Athletes rate their own competence in these areas based on a 5-point scale ranging from “not at all competent” to “extremely competent” and a composite score is calculated from their responses. The present sample demonstrated adequate internal reliability (Cronbach’s α = .82).

#### Confidence

The self-confidence subscale of the Revised Competitive State Anxiety-2 (CSAI-2R; [23]) was used to assess athletes’ confidence in sport. This measure is composed of five items that are rated on a 4-point scale ranging from “not at all” to “very much so”. The question stem was modified to target trait sport confidence instead of state sport confidence (i.e., indicate how you *generally* feel; [13]). Previous research has established factorial validity for this measure [23], which has also been used with youth populations (e.g., [24]). In the present sample, Cronbach’s α was .86.

#### Connection with coach

In the present study, the connection dimension intended to assess athletes’ relationships with both their coaches and teammates. The direct perspective of the athlete response scale from Jowett and Ntoumanis’ [25] Coach-Athlete Relationship Questionnaire (CART-Q) was used as a measure of athletes’ connection with their head coach (e.g., “I am close to my coach). The CART-Q is made up of 11 items that assess coach-athlete relationship quality using a 9-point scale ranging from “not at all” to “extremely”, and has been previously been shown to have strong psychometric properties [25]. While the CART-Q originally measured three subscales (i.e., closeness, commitment, and complementarity), these were collapsed to provide an overall measure of coach-athlete relationship quality. The CART-Q demonstrated adequate internal consistency in the present sample (Cronbach’s α = .96).

#### Connection with teammates

The Youth Sport Environment Questionnaire (YSEQ; [26]) was administered as a measure of athletes’ connection with their teammates; specifically, it assessed athletes’ perceptions of team cohesion. This instrument contains 18-items which assess athletes’ perceptions of task and social cohesion based on a 9-point scale ranging from “strongly disagree” to “strongly agree”. Factorial validity for this measure has been previously established with a large sample of youth athletes [26]. In the present sample, Cronbach’s α ranged from .89 (task cohesion) to .91 (social cohesion).

#### Character

The Prosocial and Antisocial Behavior Scale for Sport (PABSS; [27]) was used as a measure of character. The PABSS is a 20-item scale which measures the frequency in which participants engage in various types of moral behavior using a 5-point scale ranging from “never” to “very often”. This measure has shown strong psychometric properties in previous studies [27–28]. In the present study, composite measures of prosocial behavior (α = .78) and antisocial behavior (α = .82) were calculated and each demonstrated adequate reliability. The antisocial behavior items were reverse-coded such that higher scores implied less frequent antisocial behaviors.

#### Observational data

An adapted version of the Athlete Behavior Coding System (ABCS; [20]) and Observer XT software [29] were used to code the video-recorded observational data. The ABCS was originally designed to code athlete behavior in youth volleyball training sessions and intended to provide an exhaustive categorization of athlete behavior in that particular context (see [20] for additional detail on its development). The ABCS is comprised of eight main content categories: Prosocial communication, sport communication, directive communication, general communication, engaged, non-cooperative/disruptive, antisocial communication, and uncodable. The ABCS is a continuous coding system, meaning that second of athletes’ behavior during practice or competition are coded using these eight content categories. To pair with each content category, the ABCS also captures the target of each interactive behavior (e.g., coach or teammate) as well as a set of contextual codes which describe different aspects of a volleyball training session. Due to the inherent differences between volleyball training sessions and basketball competitions, some changes were made to the coding system. First, the social context dimension was replaced with a location dimension, which codes whether an athlete was on the court, on the bench, or out of view at a specific point in time. Second, minor modifications were made to the content dimension, which involved combining directive communication with its parent sport communication category, as well as refining the definitions and examples of each category to more accurately reflect the sport setting in the present study. Finally, a ball possession dimension was added to measure the frequency and duration in which each athlete has possession of the basketball during play. Due to the detailed nature of the coding process with the ABCS, a one-hour video segment for a single athlete requires 2–3 hours of coding. Thus, coding all 67 athletes in the present study required approximately 140 total hours of coding.

Using the ABCS, several measures of athlete behavior were derived for the present study (Table 1). Even though the coding process allows for the measurement of both frequency and duration, the present study focused solely on the frequency in which specific categories were activated over the course of a 40-minute game. Specifically, seven measures were the focus of analysis, which included the ball possession dimension and six specific combinations of content and target codes: Prosocial communication with coaches, prosocial communication with teammates, sport communication with coaches, sport communication with teammates, general communication with coaches, and general communication with teammates. Each measure is comprised of a content category (e.g., prosocial communication) and a target (e.g., coach) with whom that specific interaction is shared. For example, “sport communication with coaches” describes specific, individualized communication between an athlete and his/her coach. Collectively, these measures comprise the most common interactive behavior states observed across all athletes. While other content categories (e.g., antisocial communication), targets (e.g., referees), and combinations of both were coded, they were observed infrequently across a small subsample of participants, and were therefore excluded from analysis.

**Table 1:** Description of coding categories.

Category	Targets	Definition	Example
**Ball possession**	None	Athlete is clearly in possession of the basketball.	An athlete is dribbling the basketball up the court.
**Prosocial communication**	Coaches or teammates	Communication that is clearly positive in nature, and can be in response to a desirable event.	Verbal (e.g., “great job!”) and/or non-verbal (e.g., high fiving a teammate) in nature
**Sport communication**	Coaches or teammates	Communication that is related to the sport, and can be organization, technical, or tactical in nature	A coach and athlete discussing technical feedback during a time-out.
**General communication**	Coaches or teammates	Communication that is unrelated to the sport.	Two teammates talking about something that happened at school.

### Coder training and reliability

Two trained research assistants (RAs) aided the primary researcher in the adaptation of the coding system for the present study. Following an initial introduction and review of the coding system and Observer XT software, the research team engaged in several rounds of test coding whereby each individual would independently code a specific segment of video and then the RAs and primary researcher would meet to discuss any questions and compare performance. Minor refinements were made to the coding system as necessary, and this process was repeated with randomized video segments until no new issues arose. At this point, reliability testing was conducted whereby each RA was required to meet a minimum of 80% agreement on a ten-minute video segment when compared to a “gold standard” of coding completed by the primary researcher [15, 20]. The ten-minute video included 273 individual coding events, of which 218 needed to be coded correctly to reach the 80% agreement threshold. Each RA successfully reached the reliability threshold after approximately 60 hours of training, at which point one was selected based on availability to aid the primary researcher in coding videos for analysis.

### Data analysis

This study incorporated a person-centered data analysis approach, as it aimed to uncover groups of athletes with relatively homogenous developmental experiences, and then investigate potential group differences in regard to their observed behavior during competition. As such, data analysis was comprised of two main phases: 1) A cluster analysis using measures of the 4 Cs, and 2) a multivariate analysis of covariance comparing the clusters from phase 1 on measures of observed athlete behavior. All analyses were performed using SPSS version 21.

Following initial data screening, all of the questionnaires were re-scaled to a 5-point scale, which was necessary to ensure that each construct (i.e., competence, confidence, connection, and character) received equal weighting in the subsequent cluster analysis [30]. Specifically, the YSEQ (i.e., task and social cohesion) and PABSS (i.e., prosocial and antisocial behavior) were each standardized to 2.5-point scales so that the latent constructs of connection and character were weighted similarly to the other Cs. A *k*-means cluster analysis was then conducted using these 4 Cs measures in order to identify naturally occurring groups of participants. In other words, the cluster analysis grouped athletes in a way that would maximize within-group similarity and between-groups differences. A range of cluster solutions were examined and the optimal solution was determined based on their silhouette coefficients, which is used as a measure of clustering validity [31]. Follow-up independent samples *t*-tests were conducted to analyze specific differences in the 4 Cs across these clusters.

In the second phase of data analysis, the clusters of athletes were compared based on their observed behavior. After data screening, a MANCOVA was performed to assess potential group differences on each of the seven measures of observed behavior. A covariate analysis was conducted to control for the effects of gender and years of playing experience. In the event of a significant MANCOVA, follow-up tests would be conducted to determine specific group differences using a Bonferonni-corrected alpha value to adjust for multiple comparisons.

## Results

### Descriptive statistics and bivariate correlations

Means and standard deviations for all variables, in addition to bivariate Pearson correlations between all variables are shown in Table 2. Statistically significant small (*r* = +/- .3-.5) to medium strength correlations (*r* = +/- .5-.7) exist between several variables. In general, small to medium positive correlations were observed between confidence, coach-athlete relationship quality, task cohesion, and prosocial behavior. Coach-athlete relationship quality was also positively correlated with social cohesion. Among the observational variables (measures 10–16), ball possession was moderately and positively correlated with playing experience, competence, and antisocial behavior. Prosocial communication with coaches was negatively correlated with prosocial communication with teammates and positively correlated with sport communication with coaches. Prosocial communication with teammates was also positively correlated with both sport and general communication with teammates. Sport communication with coaches was also positively correlated with both sport communication with teammates and general communication with coaches. General communication with coaches and teammates were moderately, positively correlated with one another. Finally, years of playing experience was positively correlated with confidence, coach-athlete relationship quality, ball possession, and sport communication with coaches and teammates.

**Table 2:** Descriptives and correlation matrix for all questionnaire and observational variables.

Measures	M	SD	1	2	3	4	5	6	7	8	9	10	11	12	13	14
1. Playing exp,	2.73	1.96	-													
2. Competence	3.99	.78	-.02	-												
3. Confidence	3.33	.57	.35**	.21	-											
4. C-A rel. quality	5.55	1.30	.27*	-.16	.42**	-										
5. Task cohesion	5.99	1.98	.09	.22	.30*	.41**	-									
6. Social cohesion	3.93	2.07	.12	.01	-.06	.27*	.26*	-								
7. Prosoc. behavior	3.28	.74	.11	.07	.28*	.36**	.21	.17	-							
8. Antisoc. behavior	4.37	.52	-.23	-.24	-.03	.23	.20	-.03	.05	-						
9. Ball possession	23.98	16.84	.32**	.31*	.21	.03	.04	.07	.10	-.33**	-					
10. Prosoc. coach	.82	1.20	-.03	.18	.03	-.18	-.10	.12	-.15	-.13	.04	-				
11. Prosoc. team	2.85	3.19	.17	-.10	.02	.21	.14	.10	.18	.01	.13	-.29*	-			
12. Sport coach	2.68	2.53	.42**	.09	.53**	.47**	.25*	.23	.31*	-.00	.18	.26*	.17	-		
13. Sport team	2.14	2.41	.40**	.15	.12	.11	-.06	.16	.22	-.05	.31*	-.01	.33S*	.36**	-	
14. Gen. coach	.25	1.15	.18	-.06	.11	.21	.04	.07	.32*	.12	.12	.12	.21	.30*	.34**	-
15. Gen. team	4.39	5.26	.11	.11	-.05	-.08	-.18	.26*	.01	-.22	.00	.16	.30*	.15	.41**	-.10

*p < .01;

**p < .05;

Playing exp. = playing experience; C-A rel. quality = coach-athlete relationship quality; Prosoc. behavior = perceived prosocial behavior; Antisoc. behavior = perceived antisocial behavior; Prosoc. coach = prosocial communication with coach; Prosoc. team = prosocial communication with teammates; Sport coach = sport-related communication with coach; Sport team = sport-related communication with teammates; Gen. coach = general communication with coach; Gen. team = general communication with teammates.

### 4 Cs data: Cluster analysis

Data were initially screened for violations of normality, heterogeneity of variance, and the presence of outliers. Missing data were addressed using pairwise deletion, which is an acceptable approach in cluster analyses [30]. A set of *k*-means cluster analyses were conducted using the 4 Cs measures of competence, confidence, connection (i.e., coach-athlete relationship quality and task and social cohesion), and character (i.e., prosocial and antisocial behavior). Two, three, and four cluster solutions were run, and a two cluster solution emerged as optimal because it produced the highest silhouette coefficients (*m* = .31), which indicates the best fit in terms of tightness within each cluster and separation between clusters [31]. Qualitative descriptive analysis of each cluster solution also suggested that the two cluster solution presented the most theoretically interpretable solution. Screening of the data grouped by cluster revealed four potential univariate outliers. The two cluster solution was run both with and without the outliers (removed pairwise). Removal of the outliers had no effect on the cluster membership of the four outlier participants, and as such the outliers were retained. Demographic information for participants in each of the two clusters is presented in Table 3.

**Table 3:** Demographic information for participants in each cluster group.

	Cluster 1 “High Cs” (*n* = 46)	Cluster 2 “Low Cs” (*n* = 21)
Male	67.4%	71.4%
Female	32.6%	28.6%
Mean Age (SD)	12.46 (1.36)	12.33 (1.16)
Mean Years of playing experience (SD)	3.00 (2.12)	2.00 (.78)

The resultant two cluster solution was further validated using follow-up independent samples *t*-tests for each 4 Cs measure entered into the cluster analysis, with a Bonferonni-corrected alpha value of .007. Descriptive statistics for each cluster on the 4 Cs are presented in Table 4. The results of the *t*-tests showed significant differences in the scores for confidence (*t*(63) = 4.00, *p* < .001), coach-athlete relationship quality (*t*(60) = 12.62, *p* < .001), task cohesion (*t*(65) = 4.19, *p* < .001), and prosocial behavior (*t*(59) = 3.04, *p* = .004). Based on these differences, clusters 1 and 2 are hereafter labelled “high Cs” and “low Cs” respectively.

**Table 4:** Descriptive statistics by cluster on all questionnaire and observational variables.

	Cluster 1 “High Cs” (*n* = 46)	Cluster 2 “Low Cs” (*n* = 21)
	*M* (*SD*)	*M* (*SD*)
Competence (out of 5)	3.98 (.78)	4.00 (.79)
Confidence (out of 4)	3.50 (.46)*	2.95 (.62)
Coach-athlete relationship quality (out of 7)	6.25 (.64)*	3.82 (.79)
Task cohesion (out of 9)	6.60 (1.75)*	4.65 (1.84)
Social cohesion (out of 9)	4.28 (2.12)	3.14 (1.75)
Prosocial behavior (out of 5)	3.45 (.71)*	2.85 (.65)
Antisocial behavior (out of 5)	4.42 (.45)	4.26 (.65)
Ball possession	25.70 (18.77)	20.22 (11.03)
Prosocial communication with coaches	.72 (1.01)	1.03 (1.54)
Prosocial communication with teammates	3.29 (3.41)	1.91 (2.49)
Sport communication with coaches	3.38 (2.64)*	1.15 (1.35)
Sport communication with teammates	2.18 (2.55)	2.07 (2.13)
General communication with coaches	.35 (1.37)	.05 (.25)
General communication with teammates	4.48 (5.54)	4.22 (4.69)

Ball possession and communicative categories refer to the mean frequency in which each behavior was observed during a 40-minute game.

* p < .007

### Behavioral data

Basketball games ranged in length from 40 to 50 minutes due to variation in stoppages in play. To maintain consistency across observations, behavior measures were standardized to a 40 minute basketball game. Ball possession scores were also adjusted according to the amount of time each athlete spent on the court over the course of a 40 minute game. Initial data screening detected three univariate outliers which were greater than 3.29 standard deviations from the mean (sport communication with coaches, sport communication with teammates, and general communication with teammates). As such, a log(x+1) transformation was applied to all behavior variables, at which point all of the variables fell within an acceptable range. The transformed variables were used in all subsequent analyses; however, raw descriptives are presented in Table 3 for ease of interpretation. No multivariate outliers were detected using Mahalanobis distances.

A MANCOVA was conducted to examine differences between high and low Cs groups on the seven behavioral measures, while controlling for the effects of gender and playing experience. Both gender (Wilks’ λ = .44, *F*(7,57) = 10.51, *p* = .000, partial η^2^ = .56) and playing experience (Wilks’ λ = .58, *F*(7,57) = 5.89, *p* = .000, partial η^2^ = .42) showed significant main effects. There was also a significant main multivariate effect of cluster group on behavior after controlling for both gender and playing experience (Wilks’ λ = .74, *F*(7,57) = 2.81, *p* = .014, partial η^2^ = .26). Upon visual inspection of the descriptive data in Table 3, the high Cs group scored higher than the low Cs on all observed behavioral categories except prosocial communication with coaches. Follow-up analyses of variance indicated a significant difference for sport communication with coaches (*F*(1,63) = 10., *p* = .004, partial η^2^ = .14), while no other significant differences were observed for ball possession (*F*(1,63) = .18, *p* = .68, partial η^2^ = .00), prosocial communication with coaches (*F*(1,63) = .38, *p* = .54, partial η^2^ = .01), prosocial communication with teammates (*F*(1,63) = 2.24, *p* = .14, partial η^2^ = .03), sport communication with teammates (*F*(1,63) = 1.63, *p* = .21, partial η^2^ = .03), general communication with coaches (*F*(1,63) = .43, *p* = .52, partial η^2^ = .01), and general communication with teammates (*F*(1,63) = .57, *p* = .46, partial η^2^ = .01).

## Discussion

The present study explored the relationship between youth athletes’ perceptions of the 4 Cs and their observed behavior during recreational basketball games. A cluster analysis revealed two homogenous groups of athletes (i.e., high and low Cs) based on relatively high and low perceptions of confidence, coach-athlete relationship quality, task cohesion, and prosocial behavior. The findings suggested that the high Cs more frequently engaged in sport-related communication with their coaches than the low Cs. The results of this study present numerous implications for both coaching and youth development through sport, which are discussed below and broadly relate to the unique context in which the study took place.

Interestingly, the present findings provide mixed support for the previous work of Erickson and Côté [15], who conducted a longitudinal study of athlete development and coach-athlete interactions. Erickson and Côté found that coaches spent more time providing positive evaluation/encouragement (i.e., prosocial communication) and discussing mental skills with athletes (i.e., sport communication) who scored lower on the 4 Cs, and more time discussing non-sport related matters (i.e., general communication) with athletes who scored higher on the 4 Cs. While the “low” and “high” clusters described by Erickson and Côté [15] differ slightly from those in the present study, the findings from the present study demonstrated that the high Cs engaged in more frequent sport communication with their coaches. It should also be noted that in line with Erickson and Côté [15], high Cs in the present study also engaged in more frequent general (non-sport related) communication with their coaches. However, this was not significant, which may be due to the relative infrequency and wide variability of this behavior across the sample. These findings may be partly explained by considering the nature of the sport activities being observed. Erickson and Côté [15] observed volleyball training sessions, while the present study observed basketball games. Thus, while coaches may aim to provide less skilled players additional encouragement and instruction during training sessions, this effect may washout during competition when coaches are more focused on the game itself. Coaching is indeed a context-specific process, and previous research has highlighted significant differences in coaching behavior across training and competition [32]. The relative infrequency of general (non-sport related) communication with coaches (compared to sport communication) in the present study may also be explained by the supposition that during games, coaches’ behavior is primarily task-oriented (e.g., [33]). Together, these studies re-affirm the important role of the youth sport coach in relation to athlete development, and that the nature of coach-athlete relationship must be considered in light of the sport activities (e.g., training vs. competition) in which these social interactions take place [34].

The overall finding that high Cs (who are characterized in part by higher perceptions of their relationship with their coaches) engaged in more frequent sport communication with their coaches supports previous research in the area of coach-athlete relationships. It is well known that interpersonal communication is a primary channel for developing coach-athlete relationships through the transmission of trust, respect, and concern [35]. In this sense, evidence of coach-athlete interactions can be a behavioral manifestation of a high quality coach-athlete relationship. Indeed, athletes describe close and adaptive coach-athlete relationships in terms of warm, trusting and positive communication [36], and effective coaches often engage in frequent, and consistent patterns of behavior with their athletes (e.g., [37]). More broadly, these findings also provide further support for how coaches’ behavior influence PYD outcomes among athletes (e.g., [38]), as communication with coaches was associated with not only coach-athlete relationship quality, but a wide range of other PYD outcomes as well.

It is also important to remember that athletes’ perceptions and behavior are shaped by the environment in which their sport takes place. While some of the differences between training and competition have already been discussed, it is also worth considering the fact that the present study focused on a unique basketball league that was recreational in nature, but also solely exposed athletes to competitions rather than training sessions. In contrast, most other observational studies of coach and athlete behavior have investigated competitive club programs (e.g., [15, 20]) or informal sport play [21]. The basketball league’s focus on fun and equal playing time, and the observed characteristics of the high and low Cs provide support for Visek and colleagues’ [39] fun integration theory. In the present study, there were no differences across clusters in terms of performance-based indicators such as competence and ball possession. Instead, self-confidence and indicators of perceived and observed social relationships with coaches and teammates emerged as most salient. These social factors mirror many of the dimensions that Visek et al. found were most central to fun youth sport experiences (e.g., positive coaching, being a good sport, team friendships). This suggests that while the development of sport skills and competence is important, it is not a requirement for the creation of an enriching youth sport environment. Instead, sport programs for children and youth should focus on facilitating quality social relationships among both youth and adults, as social relationships are considered one of the most influential elements of the youth sport environment [40].

It is also noteworthy that athletes’ perceptions of cohesion, and in particular social cohesion, were relatively low across the entire sample. Recreational sport is generally viewed as a context for fun, enjoyment and social interaction. However, the nature of the sport environment and limited contact time may have been detrimental to the development of peer relationships and perceptions of team cohesion. Even though the present study examined a recreational basketball league, athletes’ limited contact time with teammates was during a performance-oriented activity, during which they usually arrived immediately before tip-off, and left shortly afterward. This may have suppressed the development of social cohesion due to fewer opportunities for non-sport related socialization. Indeed, Donkers, Martin, Paradis, and Anderson [41] found that task, but not social cohesion predicted enjoyment and commitment among recreational children’s soccer players. Thus, it is not surprising in the present study that both clusters showed higher perceptions of task cohesion in the present study given that teams only meet once a week to engage in a 40-minute basketball competition (a task-oriented activity). Carron and Brawley [42] posited that in sport teams, task cohesion usually develop first, followed by social cohesion, which is supported by both the present findings as well as those of Donkers and colleagues [41]. This yields key implications for youth sport programming, as these findings highlight the importance of creating opportunities for peer interactions (both task and social in nature) in order to help facilitate the development of friendships and cohesion.

The fact that neither perceptions of competence nor ball possession emerged as significantly different across clusters is also a point worthy of discussion. Indeed, past research has consistently shown a positive relationship between physical competence and positive peer relationships, as sport can act as a social currency to facilitate relationships among peers [43]. This is further supported by recent observational studies linking competence with social status in competitive youth volleyball [20], and observed peer interactions in informal sport play [21]. Again, these disparate findings may be explained in relation to the fact that the basketball league in the present study emphasized equal playing time and enjoyment for all athletes regardless of ability level. In doing so, they may have suppressed the relative importance of athletes’ competence. While non-significant, the results showed a general overall trend of the high Cs engaging in more frequent prosocial, sport, and general communication with their teammates, providing some further support for the earlier suggestion regarding the importance of facilitating social interactions among teammates.

Given the exploratory nature of this study, there are several limitations and future directions to consider. First, in this cross-sectional study athletes’ perceptions of the 4 Cs were used as a grouping variable to predict measures of observed behavior. However, the actual directionality of this relationship remains unclear. For example, do more frequent interactions with coaches lead to a stronger coach-athlete relationship, or is the opposite true? Ultimately, it is likely a reciprocal relationship in that individuals’ personal traits and sport experiences may influence their behavior, and their experiences over the course of the season may also shape their social interactions [21]. Future research should adopt longitudinal designs in order to attempt to further clarify this relationship. The inclusion of multiple observations in a longitudinal design could also enhance the validity of the observed behaviors. Second, given the inherent difficulties in observing athlete behavior in a naturalistic setting on a continuous basis, the coding system used in this study was relatively simple, focusing on the frequency in which general content categories were observed. Future studies should move beyond the sole observation of the content or “what” of athlete behavior, and also consider more nuanced aspects of such as emotional tone (e.g., [14]) or motivational climate (e.g., [44]). It would also be advantageous for follow-up studies to take advantage of emerging dynamic systems-based analytical approaches which allow for the analysis of the patterning and sequencing of observed athlete behavior [17].

Overall, the present study identified two groups of recreational youth athletes who were characterized by relatively high and low perceptions of confidence, coach-athlete relationship quality, task cohesion, and prosocial behavior. The high Cs group also engaged in more frequent sport-related communication with their coaches. Together, these findings re-affirm the importance of certain features of the coach-athlete relationship in the developmental experiences of young athletes, and highlight the consideration of the particular setting (e.g., game, practice, recreational, competitive) in which youth sport takes place.
